# Partial wrist denervation versus patient education and self-managed exercise therapy in patients with wrist osteoarthritis: study protocol for a randomized controlled trial

**DOI:** 10.1186/s13063-025-09241-7

**Published:** 2025-10-30

**Authors:** Sara L. Larsson, Tobias Tandrup, Elisabeth Brogren, Maria K. Wilcke, Elin M. Swärd

**Affiliations:** 1 Department of Clinical Science and Education, South General Hospital, Karolinska Institutet, Stockholm, Sweden; 2 Department for Hand Surgery, South General Hospital, Stockholm, Sweden; 3 Department of Hand Surgery, Skåne University Hospital, Malmö, Sweden; 4 Department of Translational Medicine, Lund University, Malmö, Sweden

**Keywords:** Osteoarthritis, Wrist, Wrist denervation, Scapho-lunate advanced collapse, SLAC, Scaphoid non-union advanced collapse, SNAC, Exercise therapy, Patient education

## Abstract

**Background:**

Wrist osteoarthritis (OA) is mainly posttraumatic, affects younger individuals than other types of OA, and can lead to significant disability. The first-line treatment is patient education and exercise, but surgery may be considered if symptoms persist. A mini-invasive surgical approach for wrist OA is partial wrist denervation, which, in theory, alleviates pain by resecting sensory nerve branches while preserving movement. Although denervation has shown mixed results, studies suggest improvements in pain and function. However, the long-term efficacy remains uncertain, with a notable percentage of patients requiring further surgical interventions. This randomized controlled trial (RCT) aims to compare the effectiveness of partial wrist denervation to nonsurgical treatment, consisting of patient education and a self-managed exercise therapy program.

**Methods:**

In this multicenter, two-armed, assessor-blinded, superiority RCT, 140 adult patients with symptomatic scapholunate advanced collapse (SLAC) or scaphoid non-union advanced collapse (SNAC) OA are randomly assigned (1:1) to receive either partial denervation through neurectomy of the anterior (AIN) or posterior (PIN) interosseous nerves or patient education combined with a self-managed exercise therapy program focused on wrist stability and muscle strength. The primary outcome is the change in the Patient-Rated Wrist Evaluation (PRWE) score at 6 months. Secondary outcomes include wrist range of motion, grip strength, pain levels, survival of the interventions, and quality of life over 12 months.

**Discussion:**

High-quality evidence regarding the effectiveness of different treatment options in wrist OA is lacking. To our knowledge, this is the first RCT comparing surgical and nonsurgical treatments in wrist OA. Using patient education and exercise therapy as a comparator to partial wrist denervation ensures ethical, clinically relevant care aligning with current OA treatment standards. Ultimately, the findings of this trial will guide and optimize treatment recommendations for wrist OA.

**Trial registration:**

ClinicalTrials.gov ID NCT06098586. Registered on 23 Oct 24.

**Supplementary Information:**

The online version contains supplementary material available at 10.1186/s13063-025-09241-7.

## Introduction

### Background and rationale {6a}

Osteoarthritis (OA) is a chronic, degenerative joint disease and a leading cause of physical disability among adults [1]. There are no treatment options available that can restore a joint damaged by OA. Instead, treatments focus on alleviating pain and enabling a return to a desired lifestyle [2]. OA in the wrist commonly affects the joints surrounding the scaphoid bone and is often caused by a degenerative or posttraumatic injury to the scapholunate ligament or an unhealed fracture of the scaphoid: scapho-lunate advanced collapse (SLAC) and scaphoid non-union advanced collapse (SNAC) [3, 4].

The least invasive surgical option in wrist OA is partial wrist denervation, which aims to reduce articular pain by resection of sensory nerve branches that innervate the wrist joint [5]. It is used either as a primary method for analgesia or combined with proximal row carpectomy (PRC), wrist fusion, or arthroplasty. In contrast to these more extensive procedures, denervation is a minimally invasive surgical technique that preserves joint motion and does not require postoperative immobilization. Studies have shown varied results following partial wrist denervation. Case series and a non-randomized trial including 12–89 participants have reported considerable improvements in patient-reported outcomes (PROMs), pain, grip strength, or motion after resection of the anterior (AIN) or posterior (PIN) interosseous nerves [6–9] or a combined AIN and PIN denervation [5, 10–12]. Conversely, a recent study investigating AIN and PIN denervation found only modest improvements in pain and patient-reported function, with uncertain clinical significance [13]. Further, a systematic review of denervation in posttraumatic wrist OA reported no significant change in pain scores and a decreased range of motion (ROM) after surgery [14]. The long-term effects of partial wrist denervation remain uncertain, as 24–28% of patients require additional surgery within 12–18 months after treatment due to persistence or recurrence of symptoms [13, 15]. Patient education and exercise therapy are recommended as core treatments for all individuals with OA, irrespective of age or symptom severity [2, 16–18]. These treatments have primarily been assessed on individuals with knee and/or hip OA with positive effects such as reduced pain and improved function [19, 20]. For hand OA in general, there is low to moderate evidence that exercise-based rehabilitation is effective for improving pain, stiffness, hand function, and grip strength in the short term [21, 22]. For wrist OA, only 1 RCT comprising 48 participants has evaluated a neuromuscular exercise therapy program as a core treatment [23]. This study found that a neuromuscular exercise program was not superior in reducing pain or improving function compared to a range of motion training program. To our knowledge, no RCTs have compared a surgical intervention to a nonsurgical control in wrist OA, and there is no high-quality evidence supporting the effectiveness of partial wrist denervation or exercise therapy for managing wrist OA.

### Objectives {7}

The aim of this study is to compare partial wrist denervation of the AIN and PIN with a nonsurgical treatment that includes patient education and a self-managed exercise therapy program. The primary objective is to investigate whether partial wrist denervation is superior to patient education and self-managed exercise therapy in improving the Patient-Rated Wrist Evaluation (PRWE) score at 6 months following the start of the intervention. Secondary objectives are to evaluate the superiority of partial wrist denervation over patient education and exercise therapy in terms of pain reduction, quality of life, objective functional outcomes, and treatment survival at the 3- and 12-month assessments.

### Trial design {8}

**Fig. 1:**
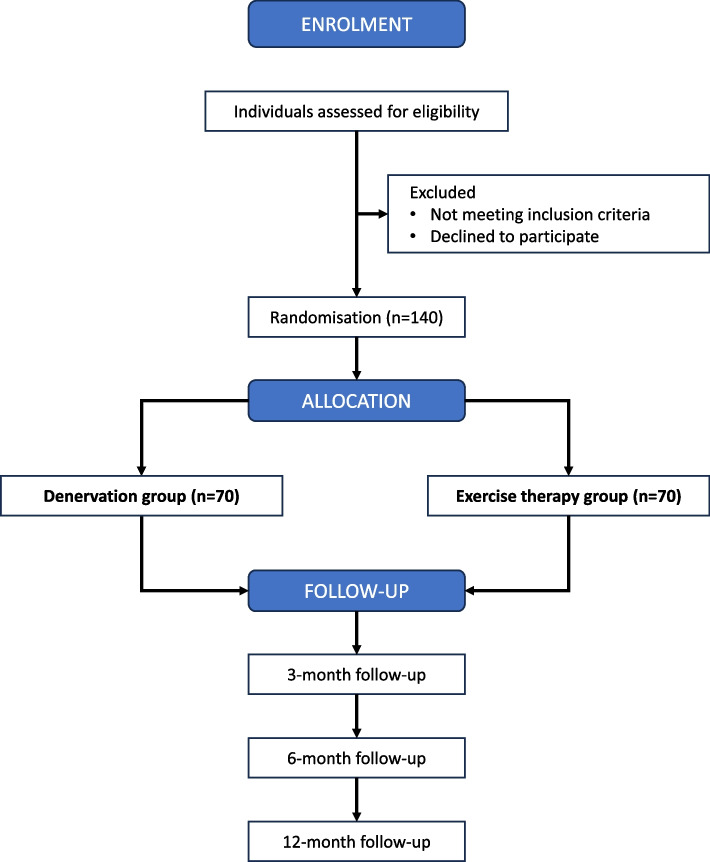
Flowchart of the trial design

**Fig. 2:**
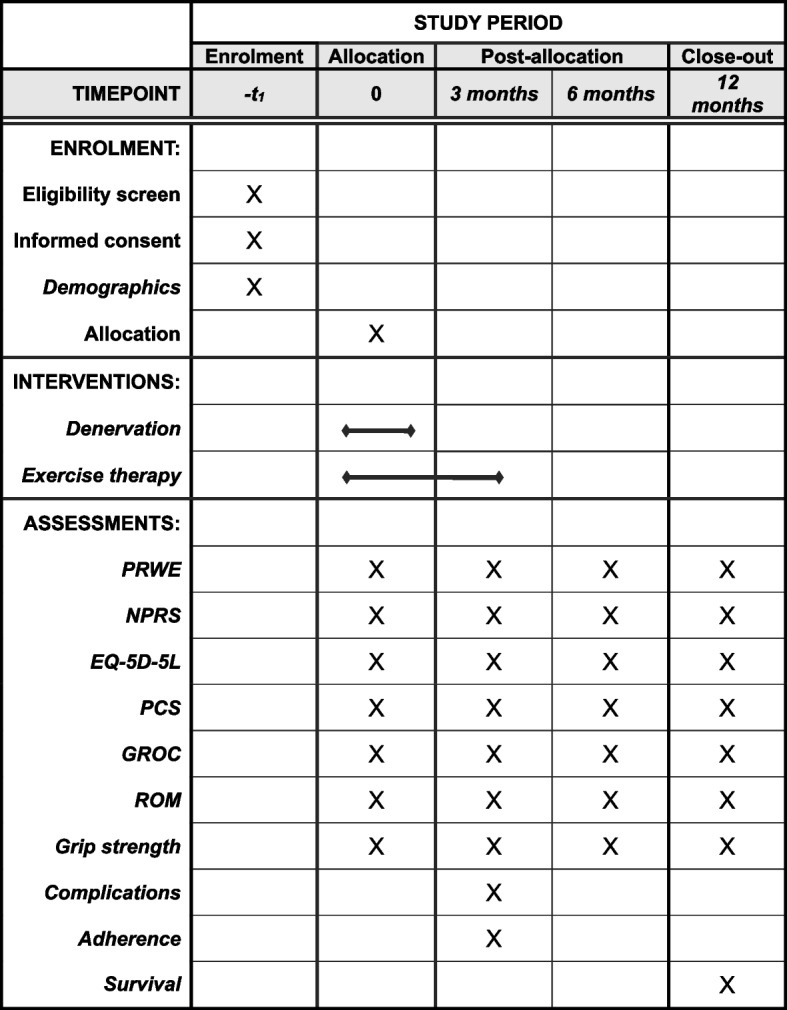
SPIRIT diagram of enrolment, interventions, and outcome measures

This is a multicentre, assessor-blinded superiority parallel-group RCT with two treatment arms, adhering to the Standard Protocol Items: Recommendations for Interventional Trials (SPIRIT) [24] and Consolidated Standards of Reporting Trials (CONSORT) guidelines [25]. The overall trial design can be found in the flowchart (Fig. 1) and the SPIRIT diagram (Fig. 2).

## Methods: participants, interventions, and outcomes

### Study settings {9}

The study will be conducted at the Department of Hand Surgery, South General Hospital, Stockholm, and the Department of Hand Surgery, Skåne University Hospital, Malmö.

### Eligibility criteria {10}

#### Inclusion criteria

- Age ≥ 18 years
- Symptomatic wrist OA (≥ 6 months), due to SLAC or SNAC, grades 1–3
- Radiological signs of osteoarthritis on posteroanterior and lateral radiographs assessed by the classification of SLAC by Watson et al. [3] and of SNAC by Vender et al. [4]. Radiographs taken 12 months prior to enrolment are eligible. If more than 12 months have passed, new radiographs will be obtained before enrolment.

#### Exclusion criteria

- Previous PIN or AIN neurectomy
- Rheumatoid arthritis (RA) or other chronic inflammatory arthritis. History of psoriasis affecting joints, gout, or pseudogout
- Symptomatic osteoarthritis in the distal radio-ulnar (DRU), scapho-trapezio-trapezoid (STT), or thumb carpometacarpal (CMCI) joints
- Ongoing infection in the hand or wrist
- Inability to co-operate with the follow-up protocol (language difficulties, severe psychiatric disorder, cognitive impairment, drug addiction)
- Intra-articular corticosteroids in the affected joint within 3 months prior to enrolment

### Who will take informed consent? {26a}

Consulting surgeons at the respective department will obtain written informed consent from all participants. The study’s purpose, methods, potential benefits, and risks will be explained. Participants can decline or withdraw their consent at any time during the study.

### Additional consent provisions for collection and use of participant data and biological specimens {26b}

N/A. This study will not involve the collection of biological specimens.

## Interventions

### Explanation for the choice of comparators {6b}

This trial will compare two different treatment concepts for patients with wrist OA: (1) Surgical treatment with partial wrist denervation of AIN and PIN and to (2) nonsurgical treatment including patient education and a self-managed exercise therapy program. We have chosen patient education and exercise therapy as a comparator, as this is considered core treatment for all individuals with OA.

### Intervention description {11a}

#### Partial wrist denervation

Surgery is conducted under local anaesthesia and bloodless field or wide-awake local anaesthesia no tourniquet (WALANT) according to the surgeon’s preference. AIN and PIN neurectomy is performed through a single dorsal incision as described by Berger [5]. Anatomical studies of the branches of the AIN have raised concerns about potential denervation of all motor branches to the pronator quadratus muscle (PQ) when the denervation technique by Berger is used [26]. To ensure that the PQ is not completely denervated, the AIN is traced distally within 2 cm of the ulnar head, and only 1 cm of the AIN is resected at this level to protect the motor function of the PQ. The skin is closed by resorbable or non-resorbable sutures according to surgeons’ preferences. Active motion of the operated joint starts immediately after surgery as tolerated by the participant. No formal hand therapy protocol is used, and participants do not receive any specific rehabilitation program (orthoses, education, exercise) after surgery, as this is standard care for this minor surgical procedure. Non-resorbable sutures are removed by a nurse at the clinic 2 weeks postoperatively, and the wound is screened for signs of infection.

#### Patient education and a self-managed exercise therapy program

The control intervention includes patient education and a revised self-managed exercise therapy program based on Larsson et al.’s protocol [23, 27]. The patient education contains information about wrist OA pathophysiology, the rationale behind exercise treatment, self-management strategies, pain management, and activity modification principles. In addition, participants are provided with a wrist orthosis along with usage instructions. The exercise therapy program aims to improve wrist stability, protect the joint, strengthen core muscles controlling the wrist, and engage the entire arm for more proximal muscle engagement [28–30]. It starts with isometric and grip-strength exercises and then introduces resistance training with a TheraBand for the elbow and shoulder while maintaining a stable wrist position, after 2 weeks. This differs from the protocol by Larsson et al., which focused solely on the affected wrist [27]. The exercise therapy program is performed at home, twice daily for 12 weeks, with follow-up assessments by the treating physiotherapist (PT) at 2, 6, and 12 weeks. A detailed description of the education and exercise therapy program can be found in Appendix 1.1.

### Criteria for discontinuing or modifying allocated interventions {11b}

To ensure that the participants can continue with the exercise therapy program without experiencing increased pain, the treating PT may need to make minor individualized adjustments to the exercise program, such as decreasing the number of repetitions or adjusting the load of the isometric exercises.

### Strategies to improve adherence to interventions {11c}

Compliance with the exercise program will be emphasized at all follow-ups, and participants will track their adherence using an exercise diary (Appendix 1.1).

### Relevant concomitant care permitted or prohibited during the trial {11d}

Intra-articular corticosteroids are allowed during follow-up but must not be administered within 3 months prior to a study assessment. Participants are allowed to continue with non-prescription analgesics as needed. The type, dosage, and frequency of analgesic use will be documented at each study assessment.

### Provisions for posttrial care {30}

Participants requiring posttrial treatment will be offered standard care for wrist OA at their respective hand surgery clinics. Those affected by treatment-related complications may seek compensation from the Swedish patient insurance system (LÖF).

### Outcomes {12}

#### Primary outcome measure

The primary outcome is the change of PRWE score [31] with a primary endpoint at 6 months following the start of the intervention. The PRWE is a wrist-specific PROM designed to assess pain and disability in daily activities over the past week. It consists of a pain subscale (5 items) and a function subscale (10 items), with a total score ranging from 0 to 100, where a higher score indicates greater disability. The PRWE is a valid and reliable tool for assessing wrist and hand injuries [32], with strong psychometric properties in wrist OA [33]. The validated Swedish version of the PRWE will be used [34].

#### Secondary outcome measures

##### Wrist range of motion (goniometer)

The range of wrist motion (flexion, extension, radial deviation, ulnar deviation, pronation, and supination) of the affected wrist will be measured with a goniometer according to standardized instructions [35].

##### Grip strength (Jamar dynamometer)

Grip strength in both hands will be assessed using a Jamar dynamometer as outlined in standardized instructions [35]. Each hand will be tested in three trials, and the mean grip strength, recorded in kilograms (kg), will be calculated for each hand.

##### The Numerical Pain Rating Scale (NPRS)

The NPRS is an 11-point Numerical Pain Rating Scale (0–10), where 0 represents no pain and 10 represents the worst pain imaginable [36]. Participants choose a value that best reflects the intensity of pain they have experienced in the affected wrist over the past week. This study will evaluate two pain measures: (1) pain at rest and (2) pain on load.

##### EQ-5D-5L (Health Quality-of-Life Measure)

The EQ-5D-5L is a standardized questionnaire created by the EuroQol Group to assess health-related quality of life (HRQoL) across five domains: mobility, self-care, usual activities, pain/discomfort, and anxiety/depression, where each question is rated on five levels [37]. A summary score from 0 to 1 is calculated based on population reference data from Sweden [38]. In addition to the five domains, the EQ-5D-5L includes the EQ Visual Analogue Scale (EQ VAS), which measures self-rated health on a 20-cm vertical scale. This scale is marked at each endpoint with the phrases “the best health you can imagine” and “the worst health you can imagine”.

##### Pain Catastrophizing Scale (PCS)

The PCS is a PROM that assesses catastrophic thinking related to pain and its potential effects on emotional and physical functioning [39]. The questionnaire comprises 13 items, each scored on a 5-point Likert scale, ranging from 0 (“Not at all”) to 4 (“All the time”). The total score can range from 0 to 52, with higher scores indicating increased levels of pain catastrophizing.

##### Global Rating of Change (GROC)

The GROC score consists of a single question asking participants to rate how their condition has changed since the start of treatment [40]. The responses are recorded on an 11-point self-report Likert scale, ranging from −5 to +5, where “−5” indicates “a very great deal worse”, “0” represents “about the same”, and “+5” signifies “a very great deal better”. In this study, the question is phrased: “Considering the problems with your wrist, how would you describe your wrist now compared to before the training period or denervation surgery?”.

#### Complications

The presence of complications to the surgical treatment, such as wound infections or wound dehiscence, will be assessed at the wound check 2 weeks postoperatively and at the 3-month study assessment.

#### Survival

The survival of the two treatments will be analysed at 12 months. Survival is the success of the treatment, indicated by the absence of additional surgery during the study period due to insufficient improvement or symptom recurrence. If a participant is scheduled for additional surgery, the date of enrolment on the waiting list for surgery is recorded, and survival, measured in months, will be calculated from the initiation of study treatment to that date.

#### Adherence to the exercise therapy program

To assess adherence, participants in the exercise therapy group will report the number of completed exercise sessions during the 12-week intervention period. After 12 weeks, they will also be asked to rate their experience with the exercise therapy program on an 11-point self-report Likert scale with the single question “How have you experienced the training program for your wrist?”, ranging from −5 to +5, where “−5” indicates “very difficult” and “ +5” signifies “very easy”.

### Participant timeline {13}

An overview of the trial design can be found in the flowchart (Fig. 1). The enrolment, intervention schedule, and outcome measures are illustrated in the SPIRIT diagram (Fig. 2).

### Sample size {14}

The reported minimal clinically important difference (MCID) of PRWE is 14 points (out of 100) based on previous research [41]. To show a difference of at least 14 points in PRWE score between partial wrist denervation and the education and exercise therapy program (SD 24) [13], after 6 months, 64 participants are required in each treatment arm. The power will be 80% (*p* < 0.05). To account for lost to follow-up, we aim to include 140 participants in total, 70 per treatment arm.

### Recruitment {15}

All eligible patients diagnosed with painful SLAC or SNAC OA at the Departments of Hand Surgery at Södersjukhuset and Skåne University Hospital will be invited to participate in the study.

## Assignment of interventions: allocation

### Sequence generation {16a}

Participants will be randomly assigned to either partial wrist denervation (intervention group) or patient education and exercise therapy (control group) with a 1:1 allocation. Sealed and unnumbered envelopes with a fixed block size of 10, with 5 envelopes per arm, will be used. The block sizes will be disclosed to the dedicated research nurse, who is blinded to the treatment allocations and randomly assigns the envelopes. The randomization will not be stratified.

### Concealment mechanism {16b}

Opaque, sealed envelopes stored in a locked safe, accessible only to the researchers responsible for the trial, will ensure allocation concealment. The sealed envelopes will be opened exclusively by the hand surgeon during the enrolment process.

### Implementation {16c}

After obtaining signed informed consent, the dedicated research nurse assigns the randomized envelope to the treating hand surgeon, who is responsible for enrolment and intervention allocation. Participants assigned to surgery are placed on the surgical waiting list by the treating hand surgeon. While the enrolling hand surgeon may perform the procedure, they will have no further contact with the participant to maintain allocation concealment. For participants assigned to patient education and exercise therapy, the hand surgeon will notify the PTs involved in the research study. The designated PTs will then contact the participant to schedule an appointment for the exercise therapy program at the clinic.

## Assignment of interventions: blinding

### Who will be blinded {17a}

Independent hand surgeons, responsible for the follow-up measurements at 3, 6, and 12 months after intervention, will remain blinded to treatment allocation. Participants in both groups will wear a Tubigrip, glove, or bandage on the wrist during all assessment visits, preventing the assessor from determining whether the participant has undergone surgery. The blinded assessor is not allowed to access the medical journal until the measurements are completed and recorded in the study protocol. After the assessment, the evaluating hand surgeon may access the medical journal, remove the Tubigrip, and discuss further treatment options with the participant, if necessary. Additionally, data analysts will also be blinded to treatment allocation.

### Procedure for unblinding if needed {17b}

We do not anticipate any circumstances that would require unblinding.

## Data collection and management

### Plans for assessment and collection of outcomes {18a}

Baseline data including demographics are collected by the hand surgeon responsible for the participant and stored in case report forms (CRF). PROMs and objective outcomes (wrist ROM and grip strength) are collected at baseline, 3, 6, and 12 months after intervention. Data on complications and the need for additional surgery or other treatments will be retrieved from the medical records.

### Plans to promote participant retention and complete follow-up {18b}

All post-enrolment appointments are provided free of charge. Participants are scheduled for regular follow-up visits to support retention. Participants who miss scheduled appointments will be contacted by phone and/or mail to reschedule. Those who are unable or unwilling to attend in-person follow-up assessments are offered the option of a video consultation. Participants who decline both in-person and video assessments are asked to complete the PROMs and return them by mail. In the event of a dropout, reasons for discontinuation will be recorded, and all data collected up to that point will be retained and analysed. Participants who withdraw after inclusion but before undergoing the intervention will be replaced. In the exercise therapy group, regular follow-ups with the treating PT will support adhering to the program and enhance retention.

### Data management {19} and confidentiality {27}

Data is handled and stored in accordance with the ethical permits and the General Data Protection Regulation (GDPR). Data from the CRFs and PROM questionnaires are transferred to secure electronic databases at the South General Hospital, Skåne University Hospital, Karolinska Institutet, and Lund University. Participants are assigned pseudonymized serial numbers, and only the researchers responsible for the trial have access to the data files and the decoding key. Data are analysed anonymously, and all results are presented at group level. To ensure data quality, researchers independent from the study team will cross-check the transferred data for errors before both interim and final analyses.

### Plans for collection, laboratory evaluation, and storage of biological specimens for genetic or molecular analysis in this trial/future use {33}

N/A. No biological specimens are collected as part of this trial.

## Statistical methods

### Statistical methods for primary and secondary outcomes {20a}

For descriptive analysis, categorical data is presented as frequencies and percentages, whereas ordinal or skewed data is presented as median (IQR) and normally distributed continuous variables as mean (SD). For the primary endpoint, comparison between partial wrist denervation, and the patient education and exercise therapy program, the Wilcoxon signed-rank test will be used for paired data, and the Mann–Whitney *U*-test will be applied for unpaired data when the data are non-normally distributed or ordinal.

*T*-tests will be employed for changes in continuous data that follow a normal distribution, while the chi-square test will be used for categorical data. Normality will be assessed by the Shapiro–Wilk test. Generalized estimating equations (GEE), adjusted for age, sex, pain catastrophizing, operated dominant hand, SLAC/SNAC OA classification according to Watson and Vender [3, 4], and treatment site (Stockholm or Malmö), will be used for analysis of differences between groups regarding longitudinal data with repeated measurements. A robust estimator covariances matrix will be used. The working correlation matrix is selected according to the lowest Quasi-likelihood under Independence Model Criterion (QICC), and linear models will be used for all variables. Kaplan–Meier analysis will be performed to generate survival curves and Cox regression to compare differences in survival at 12-month post-intervention. *P*-values < 0.05 are considered significant.

Statistical analyses will be conducted following the intention-to-treat (ITT) principle. A secondary per-protocol (PP) analysis will be performed as a sensitivity analysis to assess the robustness of the findings. If a participant requires additional wrist surgery during follow-up or has a major trauma or other disease affecting the included hand, analyses will be conducted according to ITT up to that event. Data collected after such an event will not be analysed.

### Interim analyses and stopping guidelines{21b}

Interim analyses will be performed by an independent statistician after the inclusion of 50 and 100 participants to determine whether the power calculation requires adjustment due to variance differing from initial expectations. No stopping guidelines have been established, as partial wrist denervation is a well-established treatment with a low complication rate [42]. Additionally, patient education and exercise therapy are recommended treatments for OA with minimal risk of adverse events [22].

### Methods for additional analyses (e.g. subgroup analyses) {20b}

N/A. No subgroup analyses are planned.

### Methods in analysis to handle protocol non-adherence and any statistical methods to handle missing data {20c}

Missing data will be handled and imputated according to the algorithm outlined by Jakobsen et al. [43].

### Plans to give access to the full protocol, participant-level data, and statistical code {31c}

We plan to give access to the full protocol and statistical code. For information regarding the availability of participant-level data sets, see {29}.

## Oversight and monitoring

### Composition of the coordinating centre and trial steering committee {5d}

The Department of Hand Surgery at Södersjukhuset in Stockholm and the Department of Hand Surgery at Skåne University Hospital in Malmö serve as coordination centres for this study. Both centres are responsible for overseeing and monitoring the study’s progress. The research teams comprise hand surgeons, physiotherapists, a statistician, and research nurses who are affiliated with the hand surgery departments, Karolinska Institutet, and Lund University. The principal investigator (E. S.) maintains regular communication with the on-site investigator in Malmö (EB) to oversee the study’s overall coordination and progress. There is no external trial steering committee and no endpoint adjudication committee in this study.

### Composition of the data monitoring committee and its role and reporting structure {21a}

An informal data monitoring committee, composed of designated researchers from the two Departments of Hand Surgery and independent of the study teams, will monitor the data during interim analyses.

### Adverse event reporting and harms {22}

All adverse events that may be associated with the interventions will be registered. If an adverse event occurs outside of scheduled follow-ups, participants are encouraged to contact their Department of Hand Surgery. If interim analyses reveal a high frequency of unexpected complications, early termination of the study may be considered. Study participants are covered by LÖF.

### Frequency and plans for auditing trial conduct {23}

The study teams at both coordination centres will maintain continuous communication throughout the study, discussing enrolment strategies, ethical challenges, and methodological issues. A separate auditing plan is not anticipated to be necessary for the study’s conduct.

### Plans for communicating important protocol amendments to relevant parties (e.g. trial participants and ethical committees) {25}

Significant protocol amendments which may impact on the conduct of the study will only be implemented following approval from the Swedish Ethical Review Authority. The original registration at ClinicalTrials.gov will be updated accordingly.

### Dissemination plans {31a}

The findings of this study will be published in peer-reviewed journals and shared with the participants involved and the scientific community. We also plan to present our results through scientific posters or oral presentations at relevant conferences. Data related to quality improvement of the two interventions will be communicated to healthcare professionals at other Hand Surgery Departments, county hospitals, and patient representative groups.

## Discussion

This study outlines the protocol for an RCT evaluating whether partial wrist denervation results in superior patient-reported outcomes compared to patient education and exercise therapy for individuals with symptomatic wrist OA. To our knowledge, this is the first RCT comparing surgery with a nonsurgical intervention.

Placebo-controlled RCTs are considered the gold standard and provide the highest level of evidence. This trial uses patient education and a self-managed exercise treatment program as the comparator. A systematic review and meta-analysis by Karjalainen et al. [44] suggest that trials with nonsurgical controls (no treatment, usual care, or exercise programs) generate results comparable to those with placebo (sham) controls, supporting the validity of nonsurgical controls in surgical research. Therefore, in this study, we have selected a nonsurgical control as a valid, feasible, and safe alternative to placebo (sham) surgery. Patient education combined with exercise therapy is a core treatment for OA and provides participants with at least the minimum standard of care, making the study ethically sound and clinically relevant. Additionally, using an active treatment comparator rather than sham surgery may increase patient willingness to participate, reducing selection bias. While recruitment rates in placebo-controlled trials are reported to be similar to those in nonsurgical control trials, attrition rates are notably higher in placebo-controlled trials [45]. This suggests that while placebo controls are effective in assessing the efficacy of surgical interventions, they may face challenges related to participant retention. Patient education combined with exercise therapy is a safe intervention with no reported adverse effects, whereas sham surgery introduces unnecessary risks, including scarring, infection, and anaesthesia-related complications. In addition, both partial wrist denervation and exercise therapy aim to preserve wrist motion, improve function, and reduce pain without removing osteoarthritic joint surfaces, allowing further surgical interventions if needed.

PRWE is chosen as the primary outcome since it is a valid and reliable outcome for wrist OA [33] that evaluates both function and pain and focuses on the patient’s perspective [31]. However, PROMs are inherently subjective and may be influenced by age, expectations, socioeconomic status, and psychological factors [46, 47]. To account for this, grip strength and range of wrist motion are included as objective secondary outcomes. Additionally, pain catastrophizing is incorporated as a covariate in the GEE model to adjust for psychological influences on outcomes.

The prevalence of wrist OA is relatively low compared to OA in other joints [48, 49]. By adopting a multicentre design involving two major hand surgery departments in Sweden, this trial has the capacity to recruit a sufficient sample within a reasonable time frame, ensuring statistical power and robust results. Another strength is the focus on two common types of wrist OA, SLAC and SNAC, rather than including a heterogenous group of OA types. This increases internal validity, as treatment outcomes may differ among various OA subtypes.

A potential challenge is treatment adherence in the exercise therapy group. Exercise-based interventions require adherence to the program over weeks or months to achieve benefits, which may be difficult for some individuals [50]. Since pain relief from exercise therapy may take 6–8 weeks [51], maintaining participant engagement is critical. To monitor and evaluate adherence, an exercise diary will be used.

A limitation is the lack of participant blinding due to the nature of the interventions. This introduces a risk of performance bias and detection bias, particularly for subjective outcomes [52]. To strengthen the study design, objective outcomes such as ROM and grip strength are included as secondary outcome measures. Assessors and data analysts will be blinded to minimize bias.

In conclusion, this trial aligns with the “Choosing Wisely” initiative since it includes minimal- and noninvasive treatment options that may lower the risk of low-value care, such as unnecessary and potentially harmful healthcare [53]. If partial wrist denervation proves superior to patient education and exercise therapy, it will provide an attractive surgical option for wrist OA, as it preserves motion, does not require immobilization, has a low complication rate, and is technically less demanding than other surgical salvage procedures. Conversely, if partial wrist denervation is not superior to exercise therapy, it should not be considered for treating wrist OA. Regardless of the findings, this trial will provide critical evidence for guiding and optimizing treatment recommendations for patients with SLAC and SNAC wrist OA (grades 1–3).

### Trial status

This protocol is version 2.0 (19th February 2025). Any future protocol amendments that may impact the study’s conduct or ethics require approval from the ethical review board. Recruitment of participants began in March 2024 and is estimated to be completed in 2027.

## Supplementary Information

Additional file 1: Appendix 1.1. Description of the education and exercise therapy program.

## Data Availability

Public access to participants’ data is regulated by Swedish law and the General Data Protection Regulation (GDPR) (https://www.imy.se/en/organisations/data-protection/). Therefore, the datasets from this study will not be made publicly available. However, researchers may request access by applying to the Swedish Ethical Review Authority and obtaining approval from the local data safety committee at Södersjukhuset (*GDPR.sodersjukhuset@regionstockholm.se*) and the Data Protection Officer at Region Skåne/Skåne University Hospital, tel. +46 44-309 30 00 (region@skane.se).

## Abbreviations

AIN: Anterior interosseous nerve
CMCI: Carpometacarpal I
CONSORT: Consolidated Standards of Reporting Trials
CRF: Case report forms
DRU: Distal radio-ulnar
GDPR: General Data Protection Regulation
GEE: Generalized estimating equations
GROC: Global Rating of Change
HRQoL: Health-related quality of life
ITT: Intention to treat
LÖF: Swedish patient insurance
MCID: Minimal clinically important difference
NPRS: The Numerical Pain Rating Scale
OA: Osteoarthritis
PCS: Pain Catastrophizing Scale
PIN: Posterior interosseous nerve
PQ: Pronator quadratus
RA: Rheumatoid arthritis
PRC: Proximal row carpectomy
PROM: Patient-reported outcome measure
PRWE: Patient-Rated Wrist Evaluation
PT: Physiotherapist
RCT: Randomised controlled trials
ROM: Range of motion
SLAC: Scapholunate advanced collapse
SNAC: Scaphoid non-union advanced collapse
SPIRIT: Standard Protocol Items: Recommendations for Interventional Trials
STT: Scapho-trapezio-trapezoid
WALANT: Wide-awake local anaesthesia no tourniquet
