# Supplementary material for: Partial wrist denervation versus patient education and self-managed exercise therapy in patients with wrist osteoarthritis: study protocol for a randomized controlled trial
Source: Trials. 2025 Oct 30;26:453. doi: 10.1186/s13063-025-09241-7 (PMC12577315; doi:10.1186/s13063-025-09241-7)

Wrist osteoarthritis

Patient education and exercise therapy

Skåne University Hospital and
Södersjukhuset,
Malmö and Stockholm,
Sweden

REGION
SKÅNE

Wrist osteoarthritis

Information

The wrist is a complex joint consisting of the forearm bones, eight carpal bones, and five metacarpal bones. The joint surfaces are lined with cartilage that enables smooth movement and protects against pressure. In osteoarthritis, the breakdown of articular cartilage outpaces its repair, leading to thinning and eventual loss of the cartilage. This can lead to joint pain during use and strain, which may persist even at rest. Osteoarthritis can also lead to irritation and inflammation in other parts of the joint, including the synovial membranes, ligaments, and tendons. Symptoms may include pain, stiffness, and swelling of the joint.

Wrist osteoarthritis is typically caused by previous joint or skeletal injuries. It can also result from manual labor that strains the joints and genetic factors. Osteoarthritis is a lifelong joint disease, but research on knee and hip osteoarthritis has shown that education and guided exercise therapy can alleviate osteoarthritis symptoms.

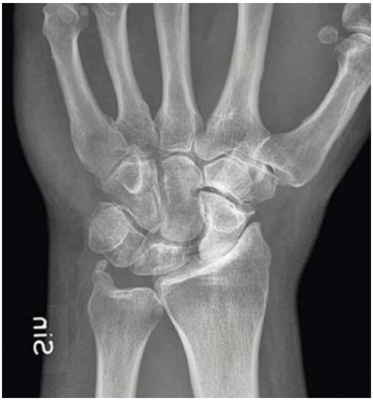

Image of an osteoarthritis affected wrist joint.

Treatment

Primary treatment with patient education and exercise therapy

There is a lot you can do yourself to reduce your wrist symptoms. The osteoarthritis-affected joint is dependent on a balanced load to function well. Every movement and use of the hand in daily activities engages the muscles to manage the load on the wrist joint to protect it. Weak muscles and poor coordination heighten the risk of joint strain. The goal of exercise therapy is to promote an active lifestyle with less pain and improved function. Exercise therapy can lead to the strengthening of the muscles controlling the joint, which can reduce the strain on the affected joint.

Neutral wrist position

The neutral wrist position is the optimal position for protecting the wrist joint. Positioning the wrist correctly provides maximum stability, reducing strain on ligaments, muscles, and tendons. Exercise therapy aims to enhance this stability, which is essential for everyday hand activities.

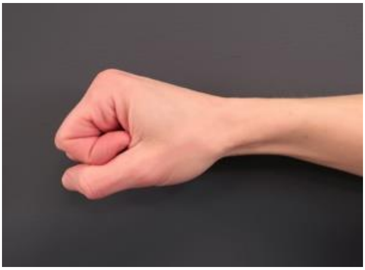
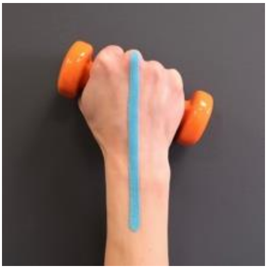

Images of the neutral wrist position.

Ergonomics and wrist support

To alleviate wrist discomfort, it is advisable to use a wrist orthosis during painful daily activities or strains. Additionally, it can be worn at night if you experience pain while resting.

It's important to prevent prolonged, painful wrist strain, recognize and adjust painful activities, and take breaks as needed.

Exercise therapy program for the wrist

Exercise therapy as a treatment for osteoarthritis is not a "quick fix"; it may take weeks or months of regular exercise before experiencing symptom relief. The exercises in the program should, therefore, be performed daily for 12 weeks. Some discomfort during exercise is acceptable, but any increase in pain should resolve within 24 hours. It's crucial to perform the movements with control and maintain the wrist in a stable, neutral position.

The aim of the exercise therapy program is to:

- Improve the strength and stability of the osteoarthritis-affected wrist joint.
- Learn to use the hand and wrist in a gentle and controlled way in everyday life.

Part 1. The exercise therapy program for the first two weeks

Perform the exercises twice a day with 10 repetitions. Hold each exercise for 5-10 seconds.

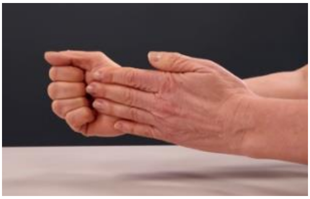

Gently push the wrist forward while resisting with the other hand.

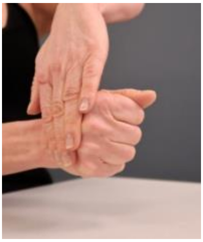

Gently push the wrist backward while resisting with the other hand.

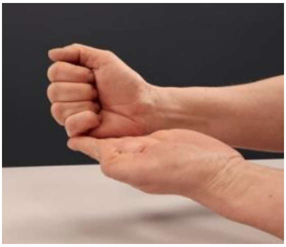

Gently push the wrist down while resisting with the other hand.

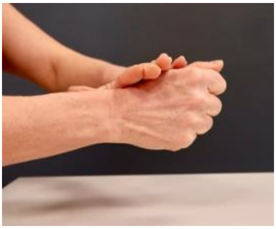

Gently push the wrist up while resisting with the other hand.

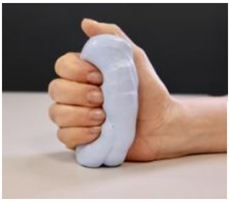

Keep your wrist in a stable neutral position and slowly squeeze the dough.

Part 2. From week 2 to week 12

Perform the exercises in Parts 1 and 2 twice a day, with 10 repetitions for each exercise.

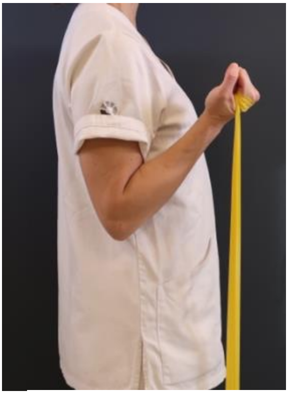
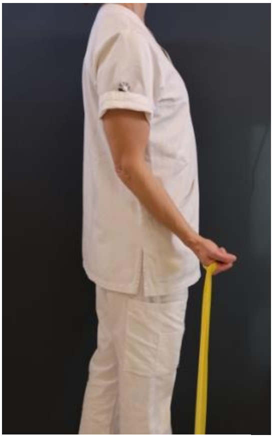

Keep your wrist in a stable neutral position throughout the movement. Flex and extend your elbow slowly with control.

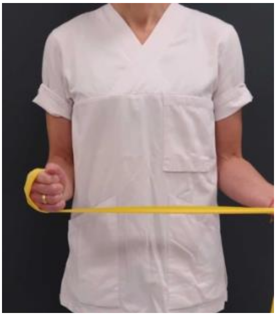
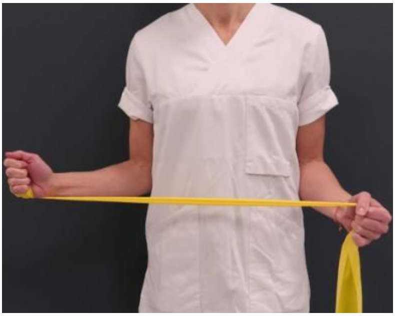

Throughout the movement, keep your wrist in a stable, neutral position. Keep your elbow flexed at 90° with your upper arm to your body. Pull your arm away from your body and back to the starting position with a slow and controlled movement.

Exercise diary

ID - Name:

To achieve the best results from your training, you should perform the exercises twice a day for a duration of 12 weeks. Please mark a tick in the box each time you complete the training. At the end of the training period, submit your exercise diary to your physiotherapist.

**Week**	**Monday**	**Tuesday**	**Wednesday**	**Thursday**	**Friday**	**Saturday**	**Sunday**
**1**	☐<br>☐	☐<br>☐	☐<br>☐	☐<br>☐	☐<br>☐	☐<br>☐	☐<br>☐
**2**	☐<br>☐	☐<br>☐	☐<br>☐	☐<br>☐	☐<br>☐	☐<br>☐	☐<br>☐
**3**	☐<br>☐	☐<br>☐	☐<br>☐	☐<br>☐	☐<br>☐	☐<br>☐	☐<br>☐
**4**	☐<br>☐	☐<br>☐	☐<br>☐	☐<br>☐	☐<br>☐	☐<br>☐	☐<br>☐
**5**	☐<br>☐	☐<br>☐	☐<br>☐	☐<br>☐	☐<br>☐	☐<br>☐	☐<br>☐
**6**	☐<br>☐	☐<br>☐	☐<br>☐	☐<br>☐	☐<br>☐	☐<br>☐	☐<br>☐

**7**	☐<br>☐	☐<br>☐	☐<br>☐	☐<br>☐	☐<br>☐	☐<br>☐	☐<br>☐
**8**	☐<br>☐	☐<br>☐	☐<br>☐	☐<br>☐	☐<br>☐	☐<br>☐	☐<br>☐
**9**	☐<br>☐	☐<br>☐	☐<br>☐	☐<br>☐	☐<br>☐	☐<br>☐	☐<br>☐
**10**	☐<br>☐	☐<br>☐	☐<br>☐	☐<br>☐	☐<br>☐	☐<br>☐	☐<br>☐
**11**	☐<br>☐	☐<br>☐	☐<br>☐	☐<br>☐	☐<br>☐	☐<br>☐	☐<br>☐
**12**	☐<br>☐	☐<br>☐	☐<br>☐	☐<br>☐	☐<br>☐	☐<br>☐	☐<br>☐

To answer after the training period of 12 weeks:

Please make a mark on the line below how you relate to the statement. Far left (-5) means "very difficult" and far right (5) means "very easy". 0 means neither hard nor easy.

How have you experienced the exercise therapy program for your wrist?

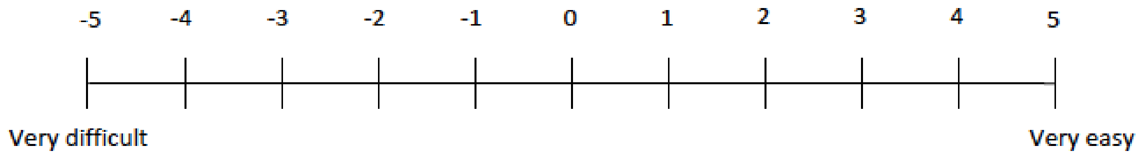

Supplement: Supplementary file 1 — Additional file 1: Appendix 1.1. Description of the education and exercise therapy program. [file 13063_2025_9241_MOESM1_ESM.pdf]
